# Supplementary material for: Enzyme-mediated aminoglycoside resistance without target mimicry
Source: Commun Chem. 2025 Aug 25;8:258. doi: 10.1038/s42004-025-01666-0 (PMC12378234; doi:10.1038/s42004-025-01666-0)
Supplement: Supplementary file 2 — Supplementary Information [file 42004_2025_1666_MOESM2_ESM.pdf]

Supplementary Information

Enzyme-Mediated Aminoglycoside Resistance Without Target Mimicry

Mark Hemmings[1,2,3], Michał Zieliński[1,2], Tolou Golkar[1,2,3], Jonathan Blanchet[1,2,3], Angelos Pistofidis[1,2,3], Kim Munro[2], T. Martin Schmeing[1,2], D. Scott Bohle[5], Albert M. Berghuis*[1,2,3,4]

[1]Department of Biochemistry, McGill University, Montréal, Québec, Canada

[2]Centre de Recherche en Biologie Structurale, McGill University, Montréal, Québec, Canada

[3]Antimicrobial Resistance Centre, McGill University, Montréal, Québec, Canada

[4]Department of Microbiology and Immunology, McGill University, Montréal, Québec, Canada

[5]Department of Chemistry, McGill University, Montréal, Québec, Canada

*** Correspondence:**

Corresponding Author

albert.berghuis@mcgill.ca

Supplementary discussion: Western blotting

To assess levels of protein translation, western blotting was attempted. Initial experiments with 6xHis-tagged fusion proteins did not yield any notable signal above background. Follow-up experiments using FLAG-tagged fusion proteins yielded the same result. We hypothesized that this was likely due to the lac promoter in the pUCP18 vector. Direct comparison of BL21 *E. coli* transformed with pUCP18 vectors against pET-15b vectors with a T7 promoter showed a clear difference in 6xHis-tagged fusion-protein expression levels. The pET-15b vectors had strong protein expression at the molecular weights corresponding to our proteins of interest (supp. Fig. 8). This was not entirely unexpected, as the pUCP18 vectors used in antibiotic susceptibility experiments have a lac promotor which does not express as highly as the T7 promoter found in the pET-15b plasmids used for crystallization [1]. Although the proteins of interest were not present in quantities suitable for detection with western blotting, they had a clear impact on bacterial antibiotic resistance. This indicates that even at a relatively low quantity, resistance enzymes are able to provide protection against antibiotics.

Supplementary methods: SDS-PAGE and western blotting

Bacterial cultures were lysed using BugBuster™ Protein Extraction Reagent (Novagen) as per manufacturer guidelines with the inclusion of cOmplete™ Protease Inhibitor Cocktail (Roche). Soluble protein concentration was determined using the Pierce BCA Protein Assay Kit (Thermo Fisher Scientific) as per the manufacturer guidelines. Proteins were separated

through sodium dodecyl sulfate-polyacrylamide gel electrophoresis (SDS-PAGE) using 15% polyacrylamide gels. The separated proteins were transferred to Immun-BLot® polyvinylidene difluoride membranes (BIO-RAD) with 0.2 μm pores that had been soaked in methanol for two minutes. The membrane was then blocked in 3% solution of bovine serum albumin (BioShop) in pH 7.4 Tris-buffered saline with TWEEN 20 (TBST): 15.23 mM Tris HCl, 4.62 mM Tris base, 150 mM NaCl, and 0.1% TWEEN 20. All antibodies used were prepared in the blocking solution. A list of all antibodies used can be found in supplementary table 3. Horseradish peroxidase (HRP)-conjugated secondary antibodies were visualized using Amersham ECL™ Western Blotting Detection Reagents (Cytiva). Blots were imaged using a BIO-RAD ChemiDoc Imaging System. Immunoblots were arranged into figures using PowerPoint (Microsoft).

Supplementary References:

1 Mierendorf, R. C., Morris, B. B., Hammer, B. & Novy, R. E. Expression and Purification of Recombinant Proteins Using the pET System. *Methods Mol Med* **13**, 257-292 (1998). https://doi.org/10.1385/0-89603-485-2:257

Supplementary figures

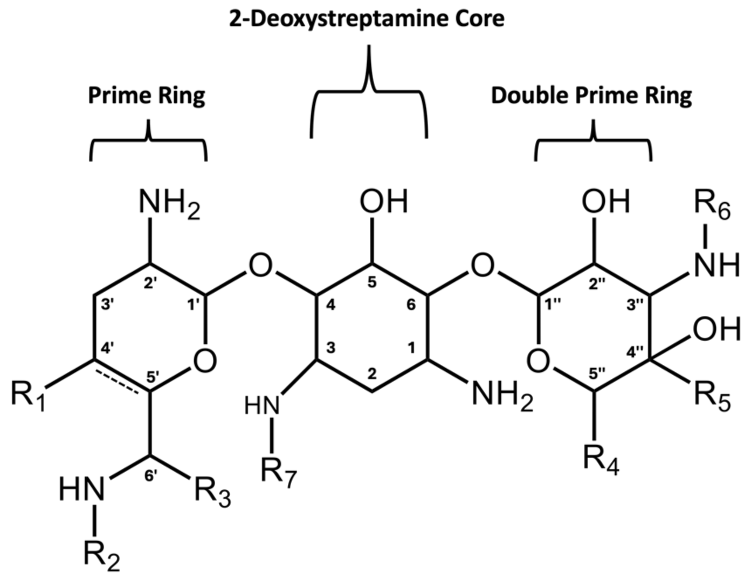

Legend	R_1	R_2	R_3	R_4	R_5	R_6	R_7
Gen C1	H	CH_3	CH_3	H	CH_3	CH_3	H
Tob	OH	H	H	CH_2OH	H	H	H
ac-Sis	H	H	H	H	CH_3	CH_3	$COCH_3$
Sis	H	H	H	H	CH_3	CH_3	H

Supplementary figure 1: Labelled diagram of generic 4,6-disubstituted aminoglycoside structure. R-groups corresponding to gentamicin, tobramycin, acetylated sisomicin, and unacetylated sisomicin.

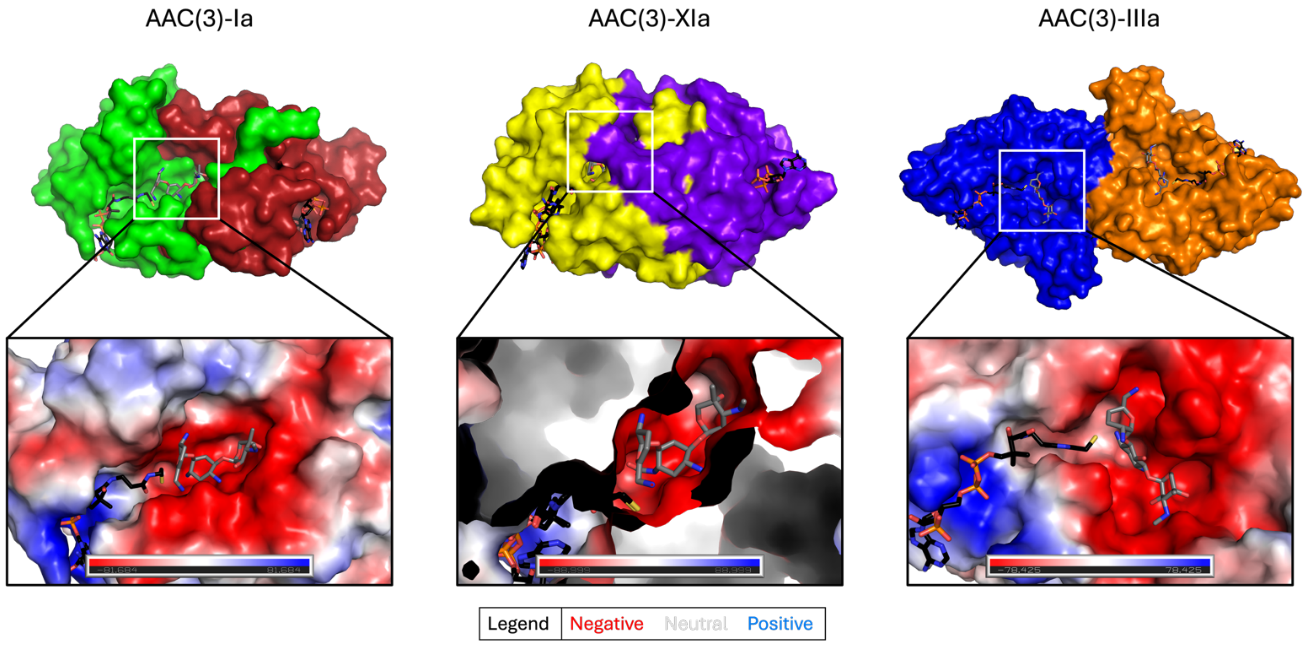

Supplementary figure 2: Visualization of electrostatic surfaces of aminoglycoside binding pockets. Electrostatic charges colored in a gradient from red (negatively charged) to blue (positively charged). Electrostatic charge was generated using Pymol protein contact potential which calculates based on a quasi-Coulombic-shaped convolution function.

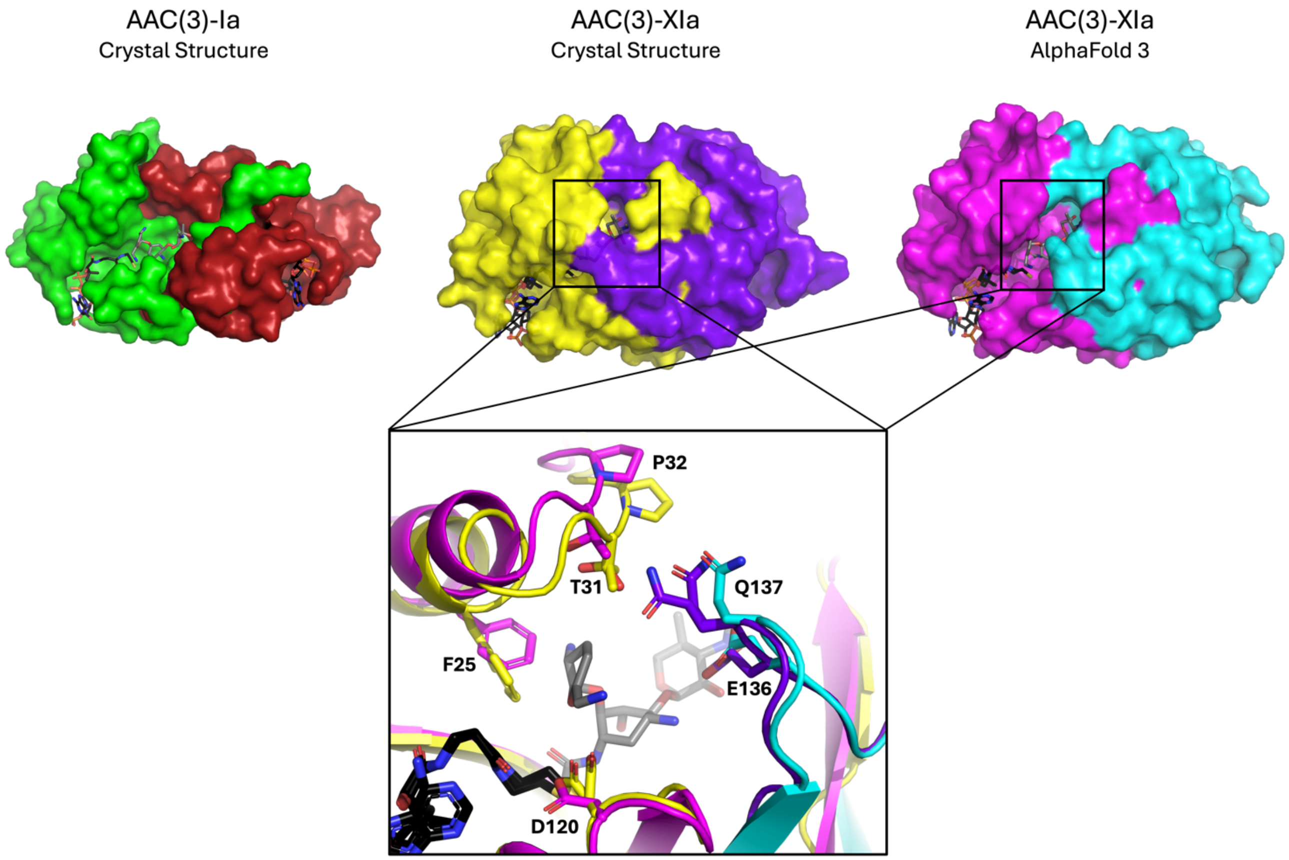

Supplementary figure 3: Comparison of aminoglycoside binding pockets. AAC(3)-Ia • sisomicin • CoASH crystal structure colored in green/red, AAC(3)-XIa • acetyl-sisomicin • CoASH crystal structure colored in yellow/purple, and AAC(3)-XIa AlphaFold 3 predicted structure colored in magenta/cyan. Since Aphafold 3 cannot yet predict the docking of our ligands of interest, acetyl-sisomicin and CoASH were superimposed from the crystal structure to better illustrate the binding pocket. Surfaces were displayed with 10% transparency to allow ligands to be seen through the closed pocket. In the magnified panel, The crystal and predicted structure of AAC(3)-XIa are overlayed. The sidechains of residues responsible for opening of the AAC(3)-XIa pocket in the AlphaFold 3 structure are shown as sticks and are labelled. Overall, differences between the AAC(3)-XIa structures are mainly the result of changes in the positions of the backbone and side chains of the loops spanning residues 30 – 35 and 136 – 137 in the tail of the adjacent protomer.

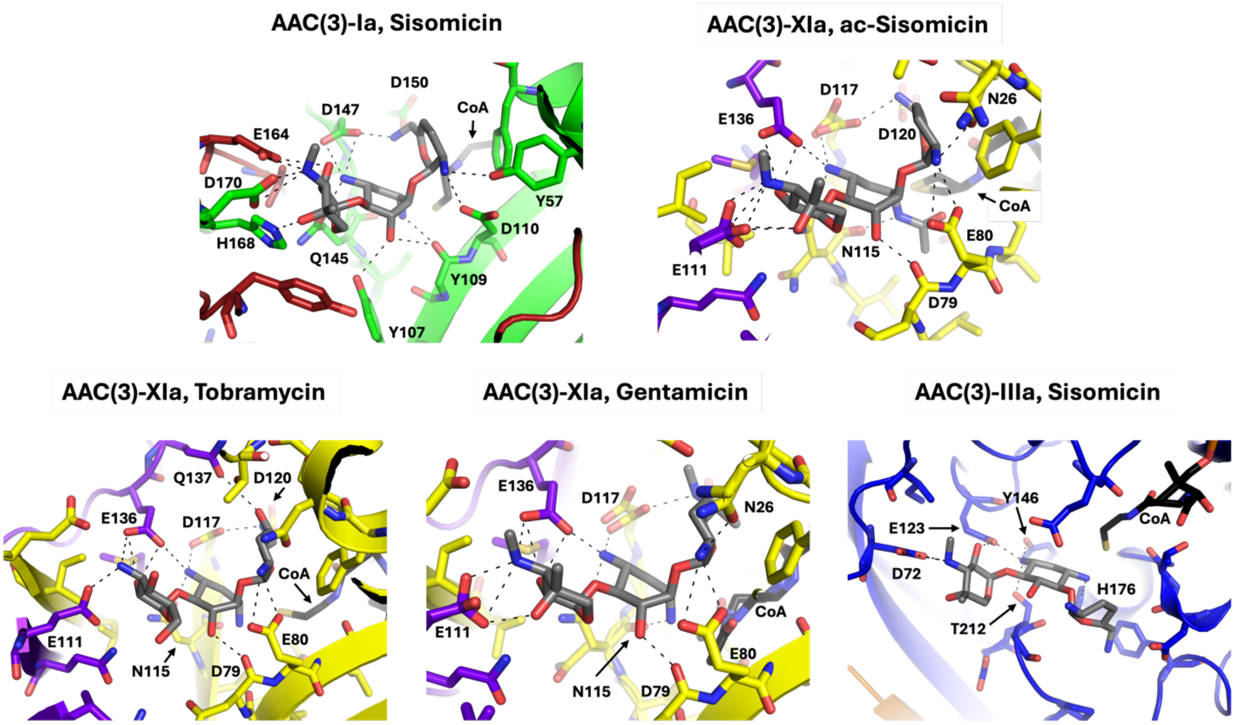

Supplementary figure 4: Hydrogen bonding networks of aminoglycoside resistance enzymes.

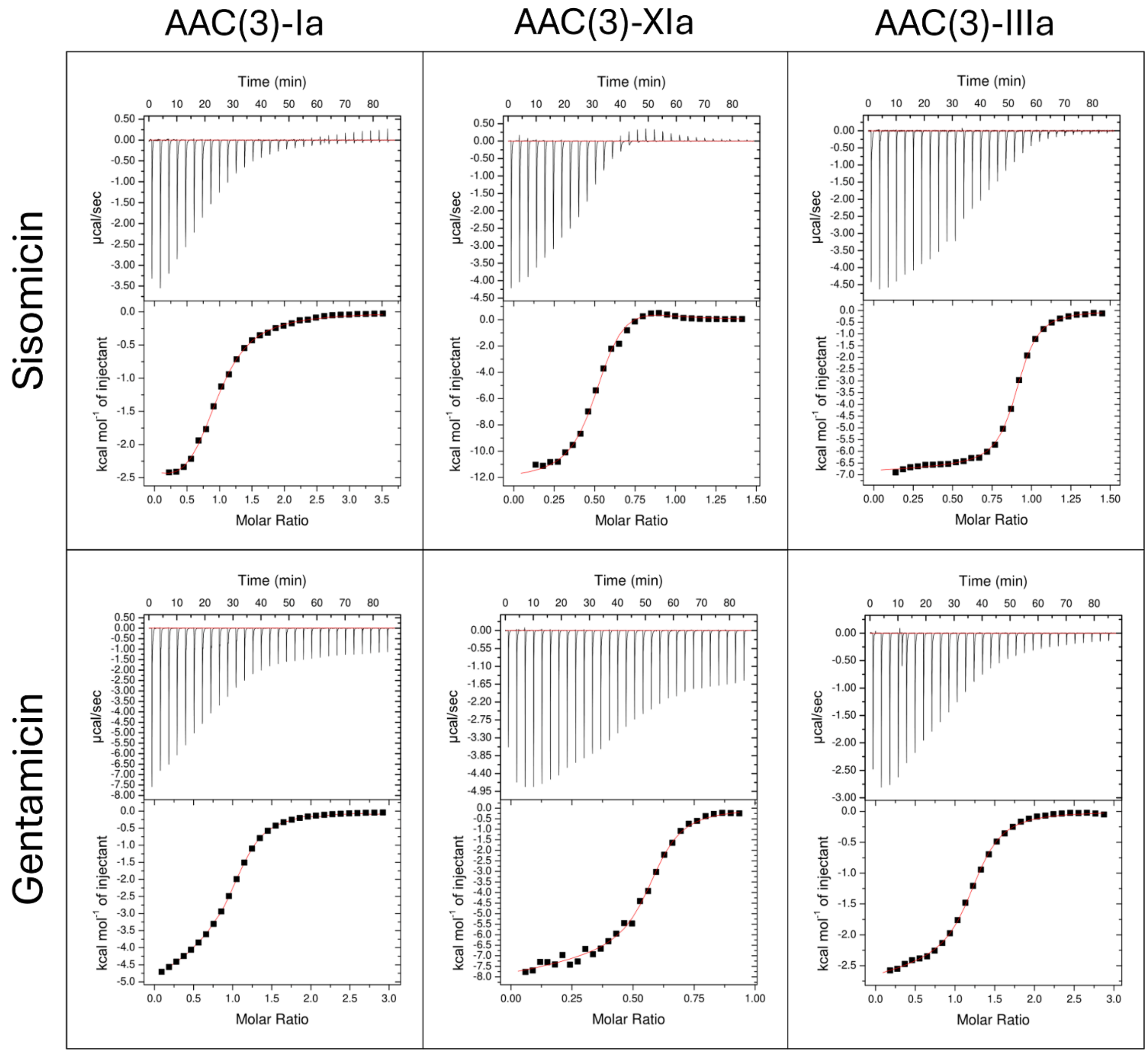

Supplementary figure 5: Representative isotherms of sisomicin and gentamicin titration into samples of AAC with CoA. Binding was monitored at 25 ˚ C across 30 injections. Upper panels contain raw calorimetric data, and lower panels contain the corresponding integrated isotherms (n = 3).

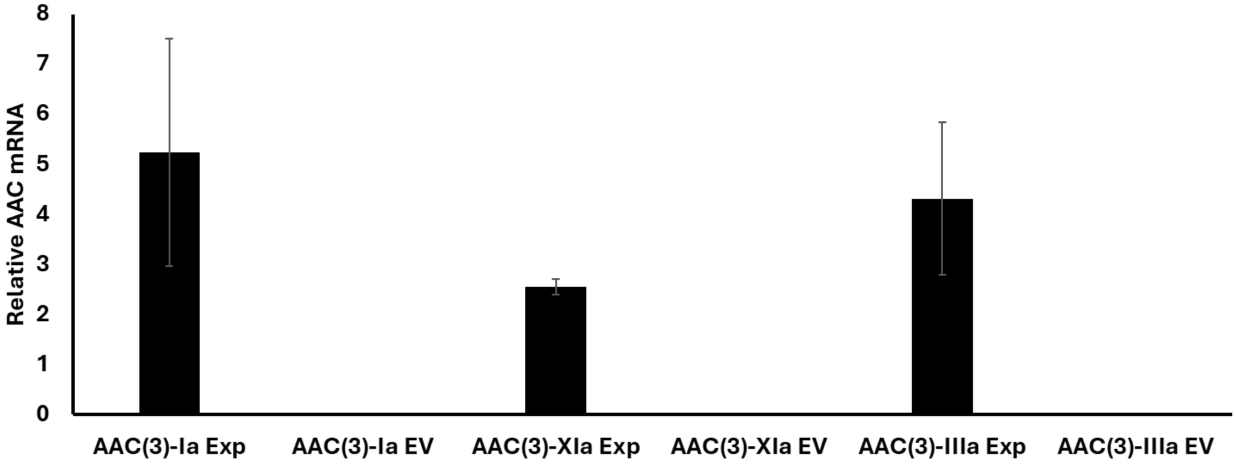

Supplementary figure 6: Relative mRNA expression of resistance genes in transformed *E. coli*. n = 3 individual experiments, relative to the expression of *hcaT* reference gene. For each resistance gene, an experimental sample of BL21(DE3) *E. coli* transformed with pUCP18 carrying the gene insert was assessed against a control sample that had been transformed with empty vector. Error bars depict the standard error of the mean. Data analyzed using a one-way ANOVA with a Tukey multiple comparison test, no significant differences between relative expression levels of experimental samples were found.

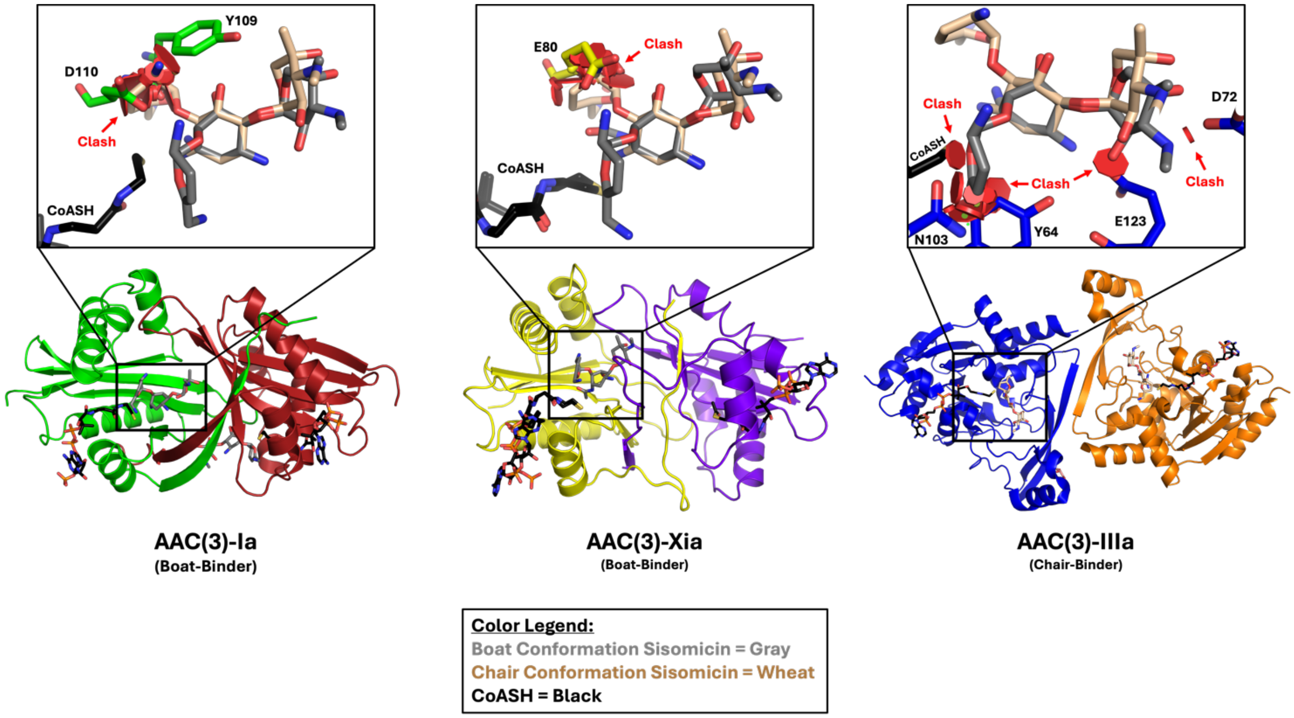

Supplementary figure 7: Modelling of non-preferred sisomicin conformations. Global view of AAC(3)-Ia, AAC(3)-XIa, and the previously published AAC(3)-IIIa in complex with sisomicin and CoASH. Cartoon representation with dimer subunits colored in green/red for AAC(3)-Ia, yellow/purple for AAC(3)-XIa, and blue/orange for AAC(3)-IIIa (PDB: 7MQK). Non-preferred orientation sisomicin molecules were aligned with crystal structure substrates through pair-fitting of 2-DOS ring atoms. The sisomicin conformation from the AAC(3)-IIIa complex was used for the chair conformation overlay in boat-binding structures and the sisomicin conformation from the AAC(3)-Ia complex was used for the boat conformation overlay in the chair-conformation structure. Boat conformation sisomicin colored in gray, chair conformation sisomicin colored in wheat brown, and CoASH in black, with non-hydrogen atoms colored in the standard red for oxygen, blue for nitrogen, and orange for phosphorus. Van der Waals overlaps shown as clash discs colored in red. Larger discs indicate greater Van der Waals overlaps.

Conformer	Relative energy (kcal/mol)
Chair ground state	0
Transition state	+10.3
Boat ground state	+7.20

Supplementary table 1: Density functional calculation energies of optimized conformers. Initial sisomicin coordinates from crystallographic structure were optimized as gas phase geometries and then PCM was used to estimate the effects of water solvation. Density functional calculations were performed with B3LPY functionals and Dunnings correlation consistent cc-pvdz basis set using Gaussian16. To estimate the conformational barriers present in the cyclohexane central ring of sisomicin, a dimethoxy substitution was used with chair and boat conformations optimized with B3LYP functionals and a cc-pvtz basis set.

Coordinates for Chair Conformation Sisomicin					Coordinates for Boat Conformation Sisomicin				
**Tag**		**X**	**Y**	**Z**	**Tag**		**X**	**Y**	**Z**
**1**	C	3.6082980	-0.9362100	-0.7169940	**1**	C	0.2960450	-1.3222790	-0.3594940
**2**	O	3.0568820	-0.0826810	0.2804840	**2**	C	-0.6805090	-1.5397110	0.8066950
**3**	C	-0.5731140	2.2258980	0.4334710	**3**	C	-0.2714840	-0.3140850	-1.3846290
**4**	N	-1.6967030	3.0711820	-0.0147490	**4**	C	-1.9595050	-2.2005610	0.2554250
**5**	C	-2.5116700	-0.4185400	-1.0680800	**5**	N	-0.1708720	-2.3257350	1.9304910
**6**	C	3.6254270	-2.3983240	-0.2104140	**6**	H	-0.9459910	-0.5480720	1.2073830
**7**	N	2.3289960	-2.7187300	0.4119340	**7**	O	1.5663270	-0.8831580	0.1605850
**8**	C	0.7864460	2.9288320	0.3629450	**8**	H	0.4587830	-2.2904410	-0.8721340
**9**	C	-3.8797690	0.2661940	-1.1449420	**9**	C	-1.8143640	-0.3451130	-1.4759870
**10**	O	-3.7663120	1.6431750	-1.4554860	**10**	O	0.2379800	-0.5465420	-2.6921400
**11**	C	4.8158480	-2.6564610	0.6817980	**11**	H	-0.0043350	0.6975210	-1.0252250
**12**	C	1.9813030	2.0472780	0.7548490	**12**	C	-2.3882990	-1.7369770	-1.1687530
**13**	N	3.1819480	2.8765230	0.7410500	**13**	H	-1.8093770	-3.2951740	0.2337320
**14**	C	-4.6910690	0.0339390	0.1444220	**14**	H	-2.7717750	-2.0143610	0.9719720
**15**	N	-6.0329590	0.6034750	-0.0601920	**15**	H	0.6088680	-1.8326360	2.3677180
**16**	C	5.8895400	-1.8632610	0.6818640	**16**	H	0.2099590	-3.2142970	1.5923930
**17**	C	1.9896890	0.7949870	-0.1431000	**17**	C	2.6978060	-1.1749830	-0.6350490
**18**	C	-4.7807860	-1.4930060	0.4275050	**18**	O	-2.3042280	0.6518430	-0.5633090
**19**	O	-5.6024280	-2.0763960	-0.5810380	**19**	H	-2.0799320	-0.0638080	-2.5091730
**20**	C	5.9877150	-0.6478530	-0.1894790	**20**	H	1.2092650	-0.4460670	-2.6343520
**21**	O	4.8967170	-0.5209640	-1.1210890	**21**	N	-2.0221570	-2.6135510	-2.2901720
**22**	C	0.6476130	0.0375310	-0.0841890	**22**	H	-3.4865420	-1.6508890	-1.1716810
**23**	O	0.6176650	-1.0263240	-1.0158270	**23**	C	3.9155230	-1.1584280	0.2857000
**24**	C	-3.3831920	-2.1217480	0.3364710	**24**	O	2.8645350	-0.2855880	-1.7244320
**25**	O	-2.6470700	-1.7913960	-0.8384810	**25**	H	2.5807970	-2.1689390	-1.0988290
**26**	C	6.1374000	0.6462590	0.6410980	**26**	C	-3.5740550	1.1939080	-0.8410570
**27**	N	6.1594920	1.8739570	-0.1504300	**27**	H	-1.0539110	-2.4666050	-2.5802220
**28**	C	-0.5730400	0.9197060	-0.3717360	**28**	H	-2.1190480	-3.5943840	-2.0265330
**29**	O	-1.7605020	0.2159480	-0.0308920	**29**	C	4.2179700	0.2578210	0.8059090
**30**	C	-5.3617430	-1.8174700	1.8136500	**30**	H	4.7782010	-1.4965770	-0.3062220
**31**	C	-6.6092460	1.2876010	1.0972070	**31**	O	3.7529900	-2.0951260	1.3413790
**32**	H	0.9445730	3.2929020	-0.6725770	**32**	C	3.1829850	1.0682730	-1.3582810
**33**	H	0.7891000	3.8221820	1.0069370	**33**	C	-3.5775200	2.6611800	-0.3704660
**34**	H	-0.7861310	1.9481670	1.4803500	**34**	O	-4.6254480	0.4335860	-0.2374820
**35**	H	1.8404440	1.7021600	1.7964000	**35**	H	-3.7794490	1.1236950	-1.9232520
**36**	H	2.1803430	1.1092420	-1.1881760	**36**	C	4.3805890	1.2124430	-0.4138520
**37**	H	-0.5728820	1.1605540	-1.4529260	**37**	N	5.4644150	0.2019200	0.5825210
**38**	H	1.0448000	-1.8007980	-0.5796010	**38**	H	3.3609730	0.5919410	1.4247370
**39**	H	-1.4330530	3.5473170	-0.8834220	**39**	H	3.4111480	1.5716480	-2.3072800
**40**	H	-1.8588330	3.8193210	0.6624090	**40**	H	2.2995500	1.5453820	-0.8998060
**41**	H	4.0091600	2.3477210	1.0144080	**41**	C	-3.4352390	2.7009080	1.1538410
**42**	H	3.3750180	3.1987900	-0.2106530	**42**	N	-2.5617590	3.4916540	-1.0110520
**43**	H	-1.9741520	-0.3260090	-2.0227970	**43**	H	-4.5686240	3.0673090	-0.6359680

**44**	H	-4.4281980	-0.2031360	-1.9768940	**44**	C	-4.9152260	0.6942950	1.0912720
**45**	H	-2.8022230	-1.8394570	1.2333790	**45**	C	4.4888350	2.6916680	-0.0111900
**46**	H	-3.4898450	-3.2156950	0.3138840	**46**	O	5.5512140	0.8181940	-1.1222320
**47**	H	-6.4200780	-1.5231620	1.8618900	**47**	C	5.4878910	1.0239440	2.7944670
**48**	H	-5.3183990	-2.9045470	1.9903900	**48**	C	-4.3767970	1.7106540	1.7828420
**49**	H	-4.8176460	-1.3077800	2.6259210	**49**	H	-2.3858630	2.4803920	1.4287520
**50**	H	-6.3045770	-1.4068250	-0.7087640	**50**	H	-3.6378870	3.7243520	1.5073640
**51**	H	-7.5533880	1.7711650	0.7994460	**51**	H	-2.7306530	3.5323310	-2.0192410
**52**	H	-6.8450290	0.5744540	1.9006440	**52**	H	-1.6566060	3.0259150	-0.9058130
**53**	H	-5.9431940	2.0659160	1.5275910	**53**	C	-5.9307510	-0.2846190	1.6258140
**54**	H	-5.9312710	1.3000650	-0.8012900	**54**	H	3.6445590	3.0187510	0.6173110
**55**	H	-3.1706840	2.0614500	-0.7882920	**55**	H	5.4239350	2.8658470	0.5396620
**56**	H	2.9976530	-0.8700690	-1.6260960	**56**	H	4.5206910	3.3221070	-0.9140410
**57**	H	5.2922000	0.6864320	1.3468860	**57**	H	6.1947000	0.6320060	-0.409515
**58**	H	7.0591600	0.5857900	1.2443560	**58**	H	6.4189000	0.8236250	3.3471530
**59**	H	5.4621600	1.7670990	-0.8924180	**59**	H	5.4774410	2.0945160	2.5446410
**60**	H	7.0546820	1.9519960	-0.6389840	**60**	H	4.6322420	0.8303120	3.4751190
**61**	H	2.2827740	-2.2509100	1.3208530	**61**	H	-4.6574630	1.8262210	2.8318410
**62**	H	2.2692700	-3.7218590	0.5974500	**62**	N	-5.5354420	-1.6962100	1.6699080
**63**	H	6.8764610	-0.7409960	-0.8417660	**63**	H	-6.8376300	-0.2155540	0.9988200
**64**	H	3.7088520	-3.0357460	-1.1090280	**64**	H	-6.2195290	0.0335240	2.6398230
**65**	H	4.7726200	-3.5407950	1.3271870	**65**	H	-5.2808660	-2.0041970	0.7293770
**66**	H	6.7394640	-2.0636150	1.3419210	**66**	H	-4.6864100	-1.7961740	2.2311560
**67**	H	0.5294510	-0.3416880	0.9506870	**67**	H	5.5842850	-0.7688800	1.8764410
**68**	H	-4.1575480	0.5324920	0.9779550	**68**	H	2.8679600	-1.9225890	1.7038250

Supplementary table 2: Sisomicin cartesian coordinates used for density functional theory calculations, as optimized by DFT calculations. Density functional calculations were performed with B3LPY functionals and Dunnings correlation consistent cc-pvdz basis set using Gaussian16.

Antibody	Supplier	Catalog No.	Host Species	Dilution
**6x-His-tag**	Invitrogen (Thermo Fisher)	MA1-21315	Mouse	1:3,000
**FLAG-tag**	Sigma-Aldrich	F1804	Mouse	1:3,000
**GAPDH**	BioLegend	631402	Rabbit	1:1,000
**Goat-anti-mouse**	Jackson ImmunoResearch	115-035-062	Goat	1:10,000
**Goat-anti-rabbit**	Jackson ImmunoResearch	111-035-046	Goat	1:10,000

Supplementary Table 3. Antibodies used for Western blotting.

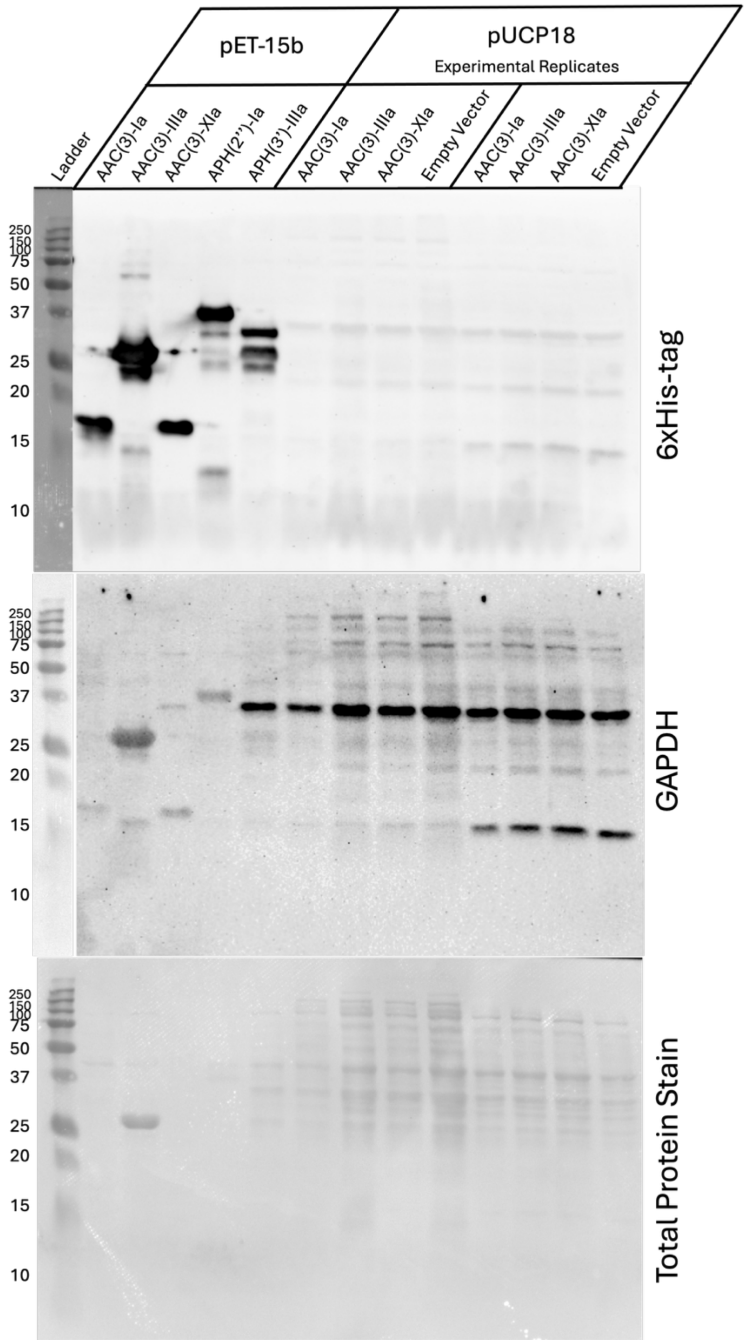

Supplementary figure 8. Comparison of protein expression between pET-15b and pUCP18 vectors. 20 ug of total protein was loaded per sample. Lysates of BL21 *E. coli* transformed with either pET-15b or pUCP18 vectors expressing AAC(3)-Ia (16.9 kDa), AAC(3)-XIa (16.4 kDa), and AAC(3)-IIIa (29.9 kDa) were compared for protein expression levels. Lysates of BL21 *E. coli* with pET-15b vectors expressing APH(2'')-Ia and APH(3')-IIIa were included as positive control and empty vector pUCP18 as a negative control. The top panel depicts 6xHis-tag probing, the middle panel GAPDH probing, and the bottom panel is total protein staining with ponceau S.
