# Supplementary material for: Enzyme-mediated aminoglycoside resistance without target mimicry
Source: Commun Chem. 2025 Aug 25;8:258. doi: 10.1038/s42004-025-01666-0 (PMC12378234; doi:10.1038/s42004-025-01666-0)
Supplement: Supplementary file 3 — Description of Additional Supplementary Files [file 42004_2025_1666_MOESM3_ESM.pdf]

Description of Additional Supplementary Files

File name: Supplementary Data 1

Description: AAC(3)-Ia CoA SIS .cif file

File name: Supplementary Data 2

Description: AAC(3)-Ia CoA .cif file

File name: Supplementary Data 3

Description: AAC(3)-XIa CoA TOY .cif file

File name: Supplementary Data 4

Description: AAC(3)-XIa CoA acSIS .cif file

File name: Supplementary Data 5

Description: AAC(3)-XIa acCoA .cif file

File name: Supplementary Data 6

Description: AAC(3)-XIa ac-amCoA Gent .cif file

File name: Supplementary Data 7

Description: AAC(3)-XIa CoA half apo .cif file

File name: Supplementary Data 8

Description: Numerical data used for figures.
